# Sirtuin 3 attenuates neuroinflammation-induced apoptosis in BV-2 microglia

**DOI:** 10.18632/aging.102375

**Published:** 2019-10-20

**Authors:** Dingzhou Zhou, Yugang Jiang

**Affiliations:** 1 Department of Neurosurgery, The Second Xiangya Hospital, Central South University, Changsha, Hunan, China

**Keywords:** Sirt3, neuroinflammation, microglial BV-2 cells, apoptosis, Mst1-JNK pathway

## Abstract

In this study, we explored the upstream regulatory mechanisms underlying inflammation-induced mitochondrial dysfunction in microglial BV-2 cells. Our results demonstrate that Sirtuin 3 (Sirt3) expression was downregulated in response to LPS-induced neuroinflammation. In addition, overexpression of Sirt3 attenuated LPS-induced BV-2 cell death. Functional studies illustrated that Sirt3 overexpression promoted normal mitochondrial function and inhibited mitochondria-dependent apoptosis in LPS-treated BV-2 cells. At the molecular level, suppressor of ras val-2 (SRV2) promoted LPS-mediated mitochondrial damage by inducing mitochondrial fission. Sirt3 overexpression, which suppressed the transcription of SRV2 and thus suppressed mitochondrial fission, played an anti-apoptotic role in LPS-treated BV-2 cells. Furthermore, Sirt3 inhibited SRV2 expression via the Mst1-JNK pathway, and re-activation of this pathway abolished the protective effects of Sirt3 on mitochondrial damage and apoptosis. Taken together, our results indicate that Sirt3-induced, Mst1-JNK-SRV2 signaling pathway-dependent inhibition of mitochondrial fission protected against neuroinflammation-mediated cell damage in BV-2 microglia. Sirt3 might therefore be an effective treatment for neuroinflammation.

## INTRODUCTION

Neuroinflammation is a pathological process that plays an important role in various acute and chronic brain disorders, including neurodegenerative disease, ischemic stroke, and traumatic brain injury [1–4]. Neuroinflammation is characterized by activation of inflammatory cascades, excessive accumulation of inflammatory cells, and increases in cytokine levels. Glial cells, which play an important role in repairing damaged brain tissue, are the primary targets of neuroinflammation [5]. Extensive neuroinflammation causes microglial cell death which in turn further augments the inflammatory response [6, 7]. Reducing inflammation-mediated microglial cell death and promoting microglial cell survival are therefore vital for stopping the progression of neuroinflammation.

Mitochondria play a central role in cell death and survival [8]. Normal mitochondria produce ATP to support cellular metabolism [9], whereas damaged mitochondria release pro-apoptotic factors to initiate programmed cell death [10]. Several studies have found that inflammation reduces mitochondrial membrane potential, promotes the opening of mitochondrial permeability transition pores (mPTPs), and increases oxidative stress [11–13]. However, the primary upstream mediator of these inflammation-induced pathological alterations in the mitochondria has not been identified. Recently, mitochondrial fission, in which individual mitochondria are divided into several compartments that can differ in membrane potential and ROS levels [14], has been identified as an early feature of mitochondrial apoptosis [15]. These fragmented mitochondria are also a primary source of pro-apoptotic proteins such as cyt-c and Smac [16, 17], which are released into the nucleus when mitochondrial apoptosis is activated. However, the effects of mitochondrial fission on neuroinflammation are largely unknown.

Several molecules capable of inducing mitochondrial fission, including Drp1, Mff, Mid49, and Fis1 have been identified [18–21]. Once activated by stress conditions, these factors work together to form a contractile ring around the outer mitochondrial membrane. Suppressor of Ras Val-2 (SRV2), which alters the balance of skeleton protein F-actin, has also recently been identified as a promoter of mitochondrial fission [22]. In this study, we examined whether inflammation-induced activation of SRV2 contributes to mitochondrial fission.

Sirtuin 3 (Sirt3), a member of the nicotinamide adenine dinucleotide-dependent histone deacetylase sub-family, has been identified as a key regulator of mitochondrial fission in several inflammation-related diseases, including high fat diet-induced hepatic inflammation [23], wound repair [24], atherosclerosis-related endothelial cell dysfunction [25], and diabetic cardiomyopathy [26]. We also reported in recent studies that pharmacological activation of Sirt3 significantly reduces the susceptibility of microglial cells to inflammation stress [27]. However, the effects of Sirt3 on mitochondrial fission in cells under inflammation conditions have not yet been experimentally examined. In this study, we investigated whether Sirt3 could attenuate neuroinflammation by modulating SRV2-induced mitochondrial fission.

## RESULTS

### Overexpression of Sirt3 attenuates LPS-mediated BV-2 cell death

**Figure 1:**
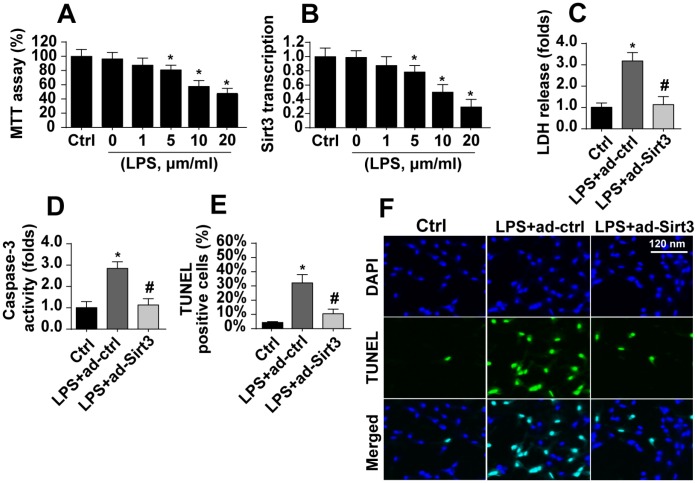
**LPS promotes BV-2 cell death by downregulating Sirt3.** (**A**) BV-2 cell viability was measured after exposure to different doses of LPS. (**B**) Sirt3 transcript levels were measured using qPCR after exposure to different concentrations of LPS. (**C**) An LDH release assay was used to evaluate cell death in response to LPS treatment. Sirt3 adenovirus was transfected into BV-2 cells to overexpress Sirt3. (**D**) Cell apoptosis was determined by analyzing the activity of caspase-3 using ELISA in BV-2 cells overexpressing Sirt3. (**E**, **F**) TUNEL staining was used to measure the cell death after exposure to LPS in BV-2 cells overexpressing Sirt3. *P<0.05 vs. control group; #P<0.05 vs. LPS+adenovirus-control group. N=3 independent experiments.

After exposure to LPS, which was used to induce neuroinflammation damage, cell viability as assessed in the MTT assay decreased dramatically (Figure 1A). Sirt3 expression also decreased rapidly at the transcriptional level (Figure 1B). These results indicated that Sirt3 downregulation and BV-2 cell damage may be linked. Adenovirus-mediated Sirt3 overexpression was used to verify the functional role of Sirt3 in neuroinflammation. As shown in Figure 1C, compared to the control group, LPS promoted release of LDH into the medium, which is indicative of cell death. Interestingly, Sirt3 adenovirus transfection largely reversed LPS-induced decreases in cell viability. In addition, ELISA indicated that caspase-3, the key promoter of cell death, was activated by LPS (Figure 1D). However, Sirt3 overexpression reduced caspase-3 activity in LPS-treated cells (Figure 1D), confirming the anti-apoptotic role of Sirt3 after neuroinflammation. Finally, the TUNEL assay was used to quantify the number of apoptotic cells. Compared to the control group, LPS increased the proportion of apoptotic cells to ~33%, while Sirt3 adenovirus transfection reduced this percentage (Figure 1E, 1F). Taken together, these results indicate that LPS-mediated BV-2 cell death could be reversed by Sirt3 overexpression.

### LPS induces mitochondrial damage in BV-2 cells

**Figure 2:**
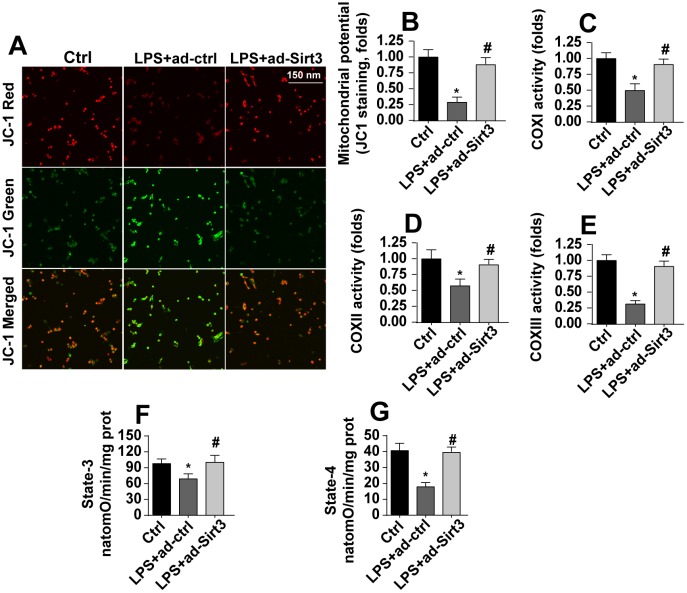
**Sirt3 overexpression attenuates LPS-mediated mitochondrial damage in BV-2 cells.** (**A**, **B**) Mitochondrial membrane potential was measured by analyzing red-to-green fluorescence intensity ratios for the JC-1 probe. (**C**, **E**) An ELISA assay was used to evaluate alterations in the mitochondrial respiratory complex of BV-2 cells after exposure to LPS stress. (**F**, **G**) Mitochondrial state-3 and state-4 respiration were measured by ELISA. BV-2 cells were treated with LPS and/or transfected with Sirt3 adenovirus. *P<0.05 vs. control group; #P<0.05 vs. LPS+adenovirus-control group. N=3 independent experiments.

At the molecular level, mitochondria have been identified as a potential target of neuroinflammation [28]. Accordingly, we explored the protective effects of Sirt3 on neuroinflammation-induced alterations in mitochondrial morphology. Under normal conditions, JC-1 probe fluorescence indicated that mitochondrial membrane potentials were generally high (Figure 2A, 2B). Interestingly, mitochondrial membrane potential was reduced after exposure to LPS (Figure 2A, 2B), as evidenced by increased green fluorescence of JC-1 probe. Transfection with Sirt3 adenovirus reversed the LPS-induced decrease in mitochondrial membrane potential (Figure 2A, 2B). ELISA was used to evaluate the activity of the mitochondrial respiratory complex, which plays a key role in the regulation of mitochondrial membrane potential. Compared to the control group, mitochondrial respiratory complex activity was reduced in response to LPS stress, and Sirt3 overexpression effectively restored mitochondrial respiratory complex function (Figure 2C–2E). By restoring mitochondrial respiratory complex activity, Sirt3 overexpression also reversed the LPS-induced decrease in mitochondrial state-3 and state-4 respiration (Figure 2F, 2G). Taken together, these results indicate that LPS impairs mitochondrial function by downregulating Sirt3.

### Mitochondrial apoptosis is inhibited by Sirt3 overexpression in LPS-treated cells

**Figure 3:**
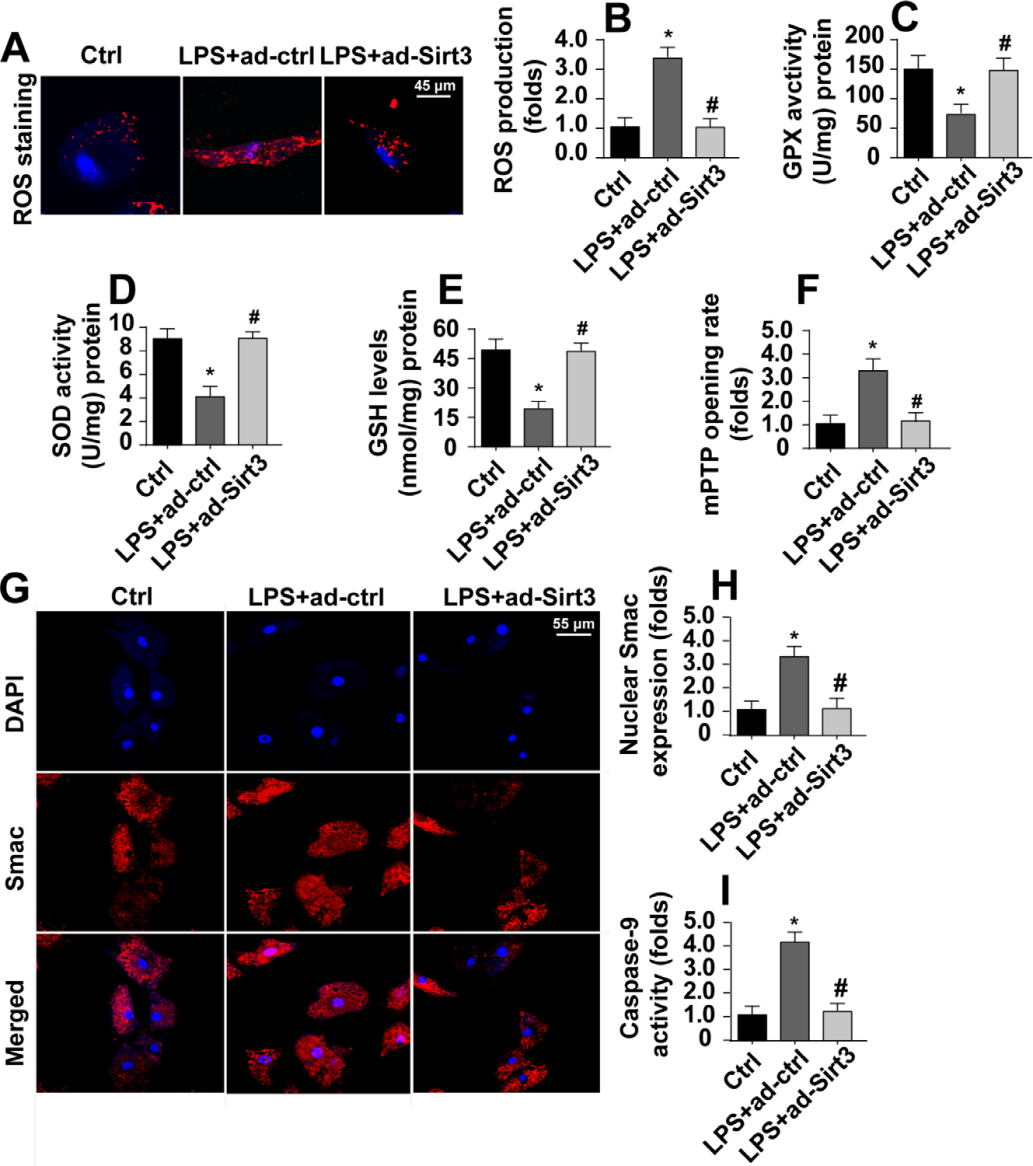
**Sirt3 inhibits LPS-induced mitochondrial apoptosis.** (**A**, **B**) ROS production was measured via immunofluorescence. BV-2 cells were treated with LPS and/or transfected with Sirt3 adenovirus. (**C**, **E**) Levels of cellular antioxidants were determined via ELISA. (**F**) mPTP opening rate was measured in response to LPS treatment and Sirt3 overexpression. (**G**, **H**) Immunofluorescence assay for Smac. Levels of nuclear Smac expression were determined in BV-2 cells treated with LPS and/or transfected with Sirt3 adenovirus. (**I**) Caspase-9 activity was detected via ELISA. BV-2 cells were treated with LPS and/or transfected with Sirt3 adenovirus. *P<0.05 vs. control group; #P<0.05 vs. LPS+adenovirus-control group. N=3 independent experiments.

Irreversible mitochondrial damage induces mitochondria-related apoptosis, which is characterized by ROS overproduction, caspase-9 activation, opening of mPTPs, and the release of pro-apoptotic factors [29]. Immunofluorescence experiments indicated that levels of ROS were significantly increased in response to LPS stress (Figure 3A, 3B). Interestingly, Sirt3 overexpression reduced ROS levels in BV-2 cells. In addition, ELISA assays also demonstrated that LPS treatment rapidly downregulated the activity of antioxidants such as SOD, GSH, and GPX (Figure 3C–3E). Sirt3 overexpression reversed this LPS-induced decrease in antioxidant levels. In addition to ROS overproduction, LPS treatment increased mPTP opening rate, and Sirt3 overexpression largely reversed this effect (Figure 3F).

Translocation of pro-apoptotic proteins, such as Smac, released from the mitochondria to the nucleus is the most important step in the activation of mitochondrial apoptosis [30]. Immunofluorescence experiments demonstrated that nuclear Smac levels increased rapidly in response to LPS (Figure 3G, 3H). Interestingly, Sirt3 overexpression significantly reduced Smac levels in the nucleus. Due to the diffusion of Smac into the nucleus, caspase-9 activity was also apparently upregulated in LPS-treated cells (Figure 3I). However, Sirt3 overexpression attenuated LPS-mediated caspase-9 activation (Figure 3I). Taken together, these results indicate that Sirt3 overexpression can block the activation of LPS-mediated mitochondrial apoptosis in BV-2 cells.

### Sirt3 overexpression reduces SRV2-associated mitochondrial fission

**Figure 4:**
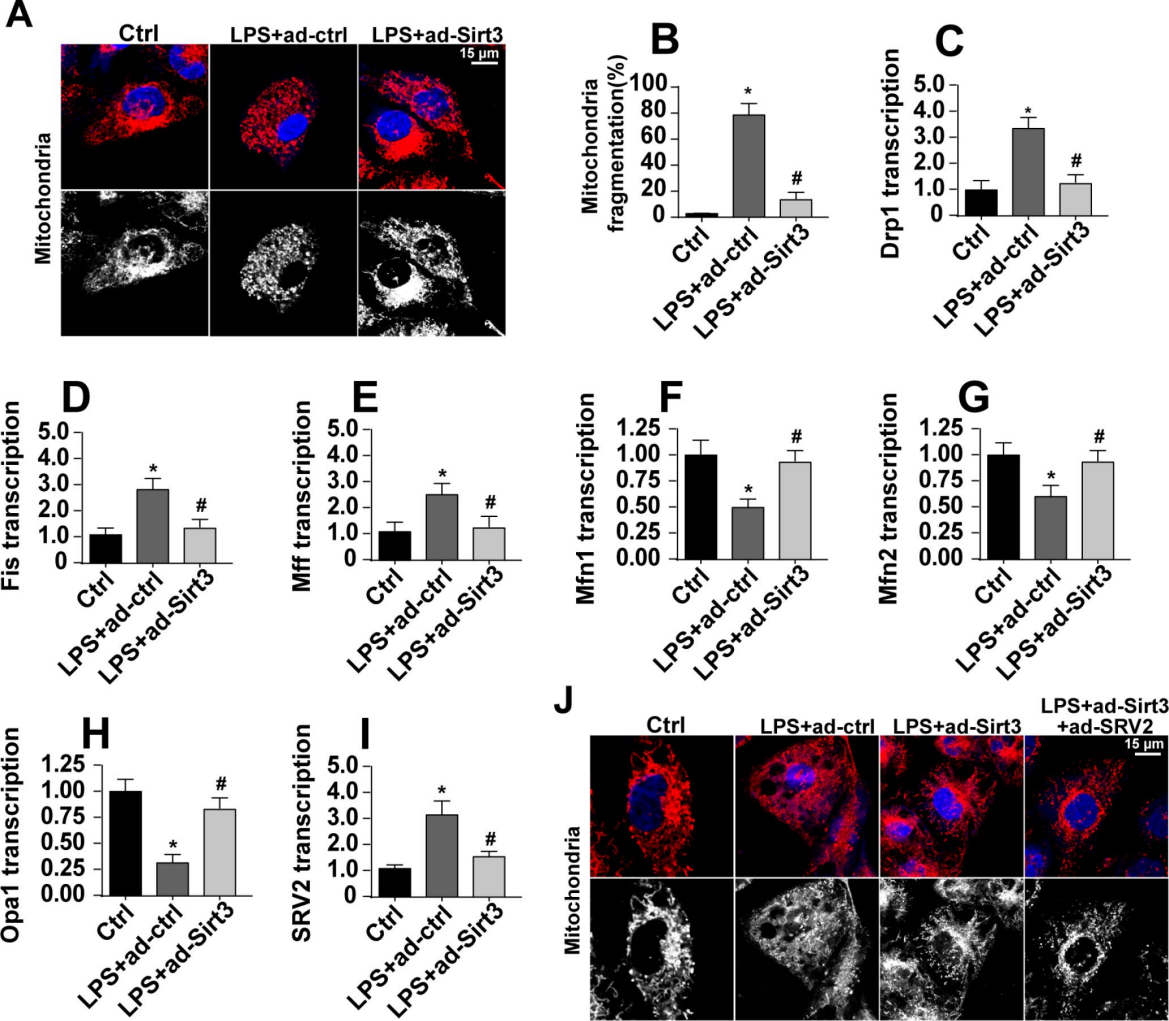
**SRV2-associated mitochondrial fission is activated by LPS and inhibited by Sirt3.** (**A**, **B**) Mitochondrial fission was measured via immunofluorescence. Numbers of fragmented mitochondria were recorded in BV-2 cells treated with LPS and/or transfected with Sirt3 adenovirus. (**C**–**H**) Transcription of mitochondrial fission-related factors. RNA was isolated from BV-2 cells treated with LPS and/or transfected with Sirt3 adenovirus. qPCR was used to measure changes in levels of mitochondrial fission-related proteins. (**I**) SRV2 expression was measured via qPCR. (**J**) Mitochondrial fission was measured in BV-2 cells transfected with SRV2 adenovirus via immunofluorescence. Numbers of fragmented mitochondria were recorded in BV-2 cells treated with LPS and/or transfected with Sirt3 adenovirus. *P<0.05 vs. control group; #P<0.05 vs. LPS+adenovirus-control group. N=3 independent experiments.

Mitochondrial fission has been identified as a novel mechanism by which mitochondrial apoptosis is initiated, and Sirt3 has been reported to inhibit mitochondrial apoptosis [31, 32]. In this study, we examined whether Sirt3 overexpression reduced mitochondrial apoptosis by repressing mitochondrial fission. First, mitochondrial fission was evaluated using immunofluorescence. As shown in Figure 4A, 4B, compared to the control group, mitochondria fragmentation was rapidly upregulated in response to LPS stress, indicating an activation of mitochondrial fission. Interestingly, Sirt3 overexpression reduced the amount of mitochondrial debris (Figure 4A, 4B). Subsequently, parameters related to mitochondrial fission were measured via qPCR. As shown in Figure 4C–4H, compared to the control group, the levels of the pro-fission factors Drp1, Fis1, and Mff were significantly elevated after exposure to LPS stress. In addition, Sirt3 adenovirus transfection significantly reduced levels of these pro-fission factors (Figure 4C–4H). In contrast, anti-fission factor levels decreased when BV-2 cells were incubated with LPS. Sirt3 adenovirus transfection rapidly increased transcription of these anti-fission factors (Figure 4C–4H). These results indicate that mitochondrial fission is activated by LPS and blocked by Sirt3.

Recently, SRV2 has been identified as a regulator of mitochondrial fission [33]. qPCR revealed that SRV2 expression was significantly elevated in LPS-treated cells and was reduced to near-normal levels after transfection with Sirt3 adenovirus (Figure 4I). To determine whether SRV2 is required for Sirt3-mediated mitochondrial fission, BV-2 cells were transfected with SRV2 adenovirus. As shown in Figure 4J, compared to the control group, LPS-induced mitochondrial fragmentation was reversed by Sirt3 overexpression. However, SRV2 adenovirus transfection abolished the inhibitory effects of Sirt3 on mitochondrial fission (Figure 4J). Taken together, these results confirm that SRV2 is essential for Sirt3-induced reduction of mitochondrial fission in LPS-treated BV-2 cells.

### Activation of mitochondrial fission abolishes the protective effects of Sirt3 overexpression in mitochondria

**Figure 5:**
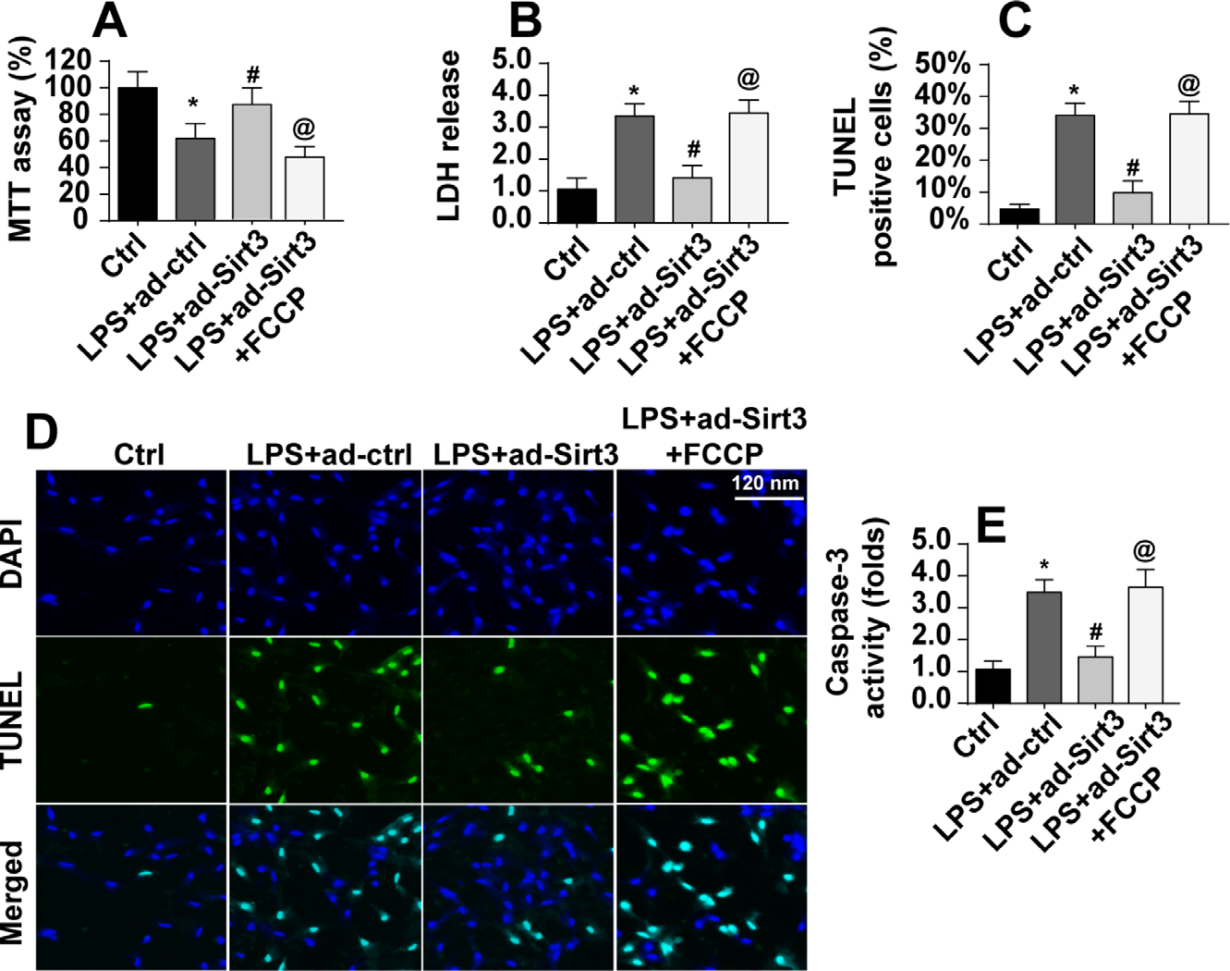
**Re-activation of mitochondrial fission abolished the protective effects of Sirt3 on BV-2 cell survival in the presence of LPS.** (**A**) Cellular viability was measured via MTT assay. FCCP was administered to re-activate mitochondrial fission in Sirt3-overexpressing cells. (**B**) An LDH release assay was used to evaluate cell damage in BV-2 cells treated with LPS and/or transfected with Sirt3 adenovirus; FCCP was used to re-activate mitochondrial fission in Sirt3-overexpressing cells. (**C**, **D**) TUNEL staining was used to measure cell death after exposure to LPS. FCCP was used to reactivate mitochondrial fission. (**E**) Cell apoptosis was examined by measuring caspase-3 activity via ELISA. *P<0.05 vs. control group; #P<0.05 vs. LPS+adenovirus-control group; @P<0.05 vs. LPS+adenovirus-Sirt3 group. N=3 independent experiments.

Next, we examined whether mitochondrial fission was critical for LPS-induced apoptosis in BV-2 cells. First, cell viability and apoptosis were assessed in Sirt3-overexpressing BV-2 cells after administration of an activator of mitochondrial fission. As shown in Figure 5A, compared to the control group, LPS-induced reductions in cell viability were reversed by Sirt3 overexpression, while activation of mitochondrial fission reversed this effect. In addition, cell damage as assessed in an LDH release assay was increased by LPS and reduced by Sirt3 (Figure 5B). Interestingly, the protective effects of Sirt3 were blocked by the mitochondrial fission activator. TUNEL staining was used to quantify the number of apoptotic cells. As shown in Figure 5C, 5D, Sirt3 inhibited LPS-induced cell apoptosis, and mitochondrial fission activation reversed this effect. In addition, caspase-3, the key promoter of apoptotic signaling, was activated after exposure to LPS stress. Although Sirt3 adenovirus repressed LPS-mediated caspase-3 activation, this effect was abolished after re-activation of mitochondrial fission (Figure 5E). In sum, these data suggest that Sirt3 protects BV-2 cells against LPS-mediated apoptosis by repressing mitochondrial fission.

### Sirt3 affects SRV2 via the Mst1-JNK pathway

**Figure 6:**
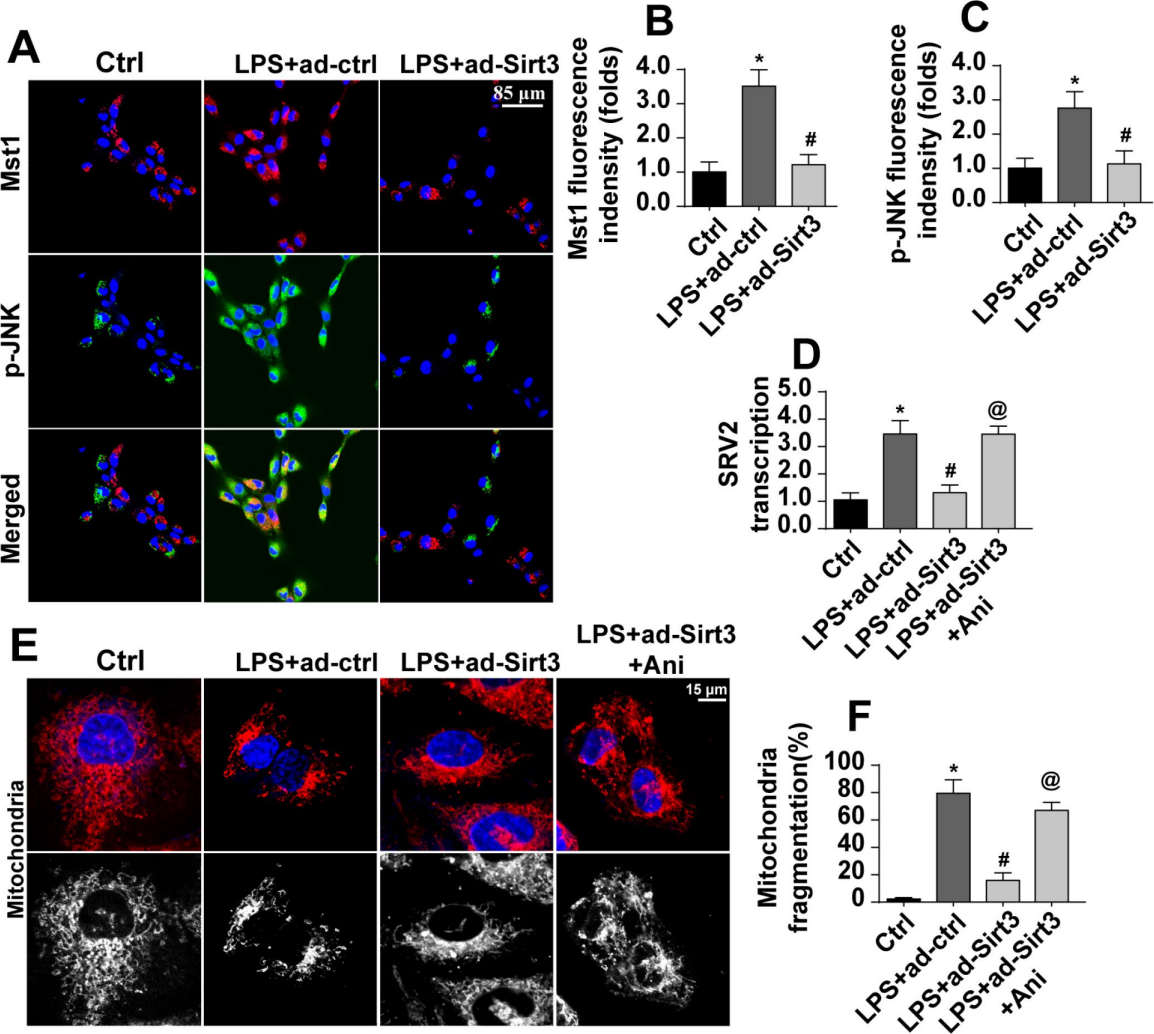
**Sirt3 modulates SRV2-associated mitochondrial fission via the Mst1-JNK pathway.** (**A**–**C**) Immunofluorescence assay for Mst1 and p-JNK. BV-2 cells were treated with LPS and/or transfected with Sirt3 adenovirus. (**D**) RNA was isolated from BV-2 cells treated with LPS and/or transfected with Sirt3 adenovirus. qPCR was then used to measure changes in SRV2 levels. Ani, an agonist of the Mst1-JNK pathway, was used to re-activate its activity. (**E**, **F**) Mitochondrial fission was measured via immunofluorescence. Numbers of fragmented mitochondria in BV-2 cells treated with LPS and/or transfected with Sirt3 adenovirus were recorded. Ani was used to activate the Mst1-JNK pathway. *P<0.05 vs. control group; #P<0.05 vs. LPS+adenovirus-control group; @P<0.05 vs. LPS+adenovirus-Sirt3 group. N=3 independent experiments.

The above data illustrated the mechanism by which Sirt3 preserves mitochondrial function and BV-2 cell viability in the presence of LPS. However, how Sirt3 affected SRV2 in LPS-treated BV-2 cells remained unclear. Our previous studies and other recent experiments have identified the Mst1-JNK axis as an important signaling pathway for neuroinflammation and inflammation-induced neuron death [34]. We therefore examined whether this pathway was activated by Sirt3 and contributed to SRV2 modification under LPS stress. Firstly, immunofluorescence was used to verify that Mst1 and JNK expression were altered in the presence of LPS. As shown in Figure 6A–6C, little Mst1 and JNK expression was observed in normal BV-2 cells. Interestingly, Mst1 and JNK levels increased rapidly after exposure to LPS and were reduced to near-normal levels after transfection with Sirt3 (Figure 6A–6C), indicating that the Mst1-JNK pathway was activated by LPS and inactivated by Sirt3 overexpression. To understand the role of the Mst1-JNK pathway in the pathogenesis of neuroinflammation, SRV2 transcription was measured in Sirt3-overexpressed cells treated with an Mst1-JNK pathway agonist. As shown in Figure 6D, compared to the control group, SRV2 transcription was upregulated by LPS stress. Although Sirt3 adenovirus repressed LPS-mediated SRV2 activation, reactivation of the Mst1-JNK pathway counteracted this effect. As shown in Figure 6E, 6F, immunofluorescence indicated that, compared to the control group, Sirt3 adenovirus transfection attenuated LPS-induced mitochondrial fragmentation; this inhibitory effect of Sirt3 was eliminated upon re-activation of Mst1-JNK pathway. Together, these results indicated that Sirt3 affects SRV2 expression via the Mst1-JNK pathway.

### Re-activation of the Mst1-JNK pathway impairs Sirt3-mediated mitochondrial protection

**Figure 7:**
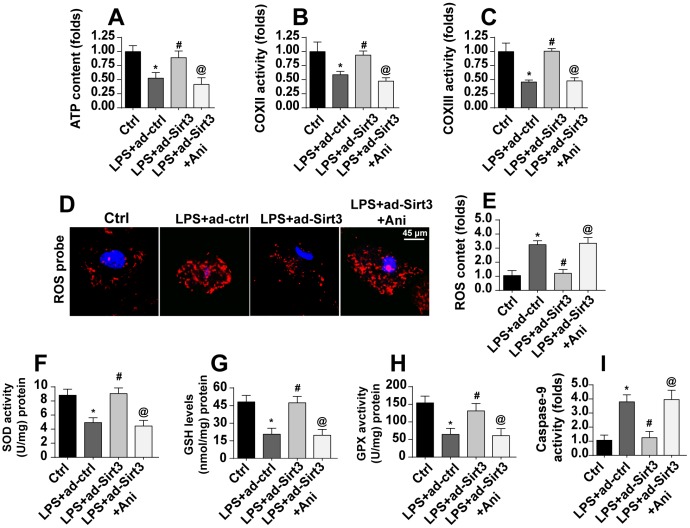
**Activation of the Mst1-JNK pathway attenuates Sirt3-meidated mitochondrial protection.** (**A**) ATP production was measured via ELISA. BV-2 cells were treated with LPS and/or transfected with Sirt3 adenovirus. Ani was used to activate the Mst1-JNK pathway. (**B**, **C**) ELISA was used to evaluate alterations in the mitochondrial respiratory complex in BV-2 cells after exposure to LPS stress. (**D**, **E**) ROS production was measured via immunofluorescence. BV-2 cells were treated with LPS and/or transfected with Sirt3 adenovirus. (**F**–**H**) Cellular antioxidant levels were determined via ELISA. I. Caspase-9 activity was detected via ELISA. *P<0.05 vs. control group; #P<0.05 vs. LPS+adenovirus-control group; @P<0.05 vs. LPS+adenovirus-Sirt3 group. N=3 independent experiments.

A final set of experiments was conducted to verify whether Sirt3-mediated mitochondrial protection was controlled by the Mst1-JNK pathway. Total ATP production, for which mitochondria are solely responsible, was reduced by LPS and restored to near-normal levels by Sirt3 overexpression. Interestingly, an Mst1-JNK pathway agonist caused a dramatic decline in ATP production in Sirt3-overexpression cells (Figure 7A). LPS-mediated inactivation of the mitochondrial respiratory complex was reversed by Sirt3 adenovirus, and this effect was blocked upon re-activation of the Mst1-JNK pathway (Figure 7B, 7C).

Mitochondrial apoptosis was also measured in these experiments. LPS induced oxidative stress as indicated by high ROS levels in BV-2 cells (Figure 7D, 7E). Although Sirt3 overexpression reduced generation of ROS, this anti-oxidative effect was abolished in cells treated with an Mst1-JNK pathway agonist (Figure 7D, 7E). Similarly, antioxidant activity was sustained by Sirt3 in the presence of LPS and decreased upon re-activation of Mst1-JNK pathway (Figure 7F–7H). Finally, caspase-9, a marker of mitochondrial apoptosis, was activated by LPS and reduced in Sirt3-overexpression cells (Figure 7I). Interestingly, the Mst1-JNk pathway agonist abolished the anti-apoptotic effects of Sirt3 on mitochondria. Taken together, our results demonstrate that Sirt3 protected mitochondria against LPS stress by inhibiting the Mst1-JNK axis.

## DISCUSSION

From an epidemiological point of view, neuroinflammation is increasingly considered an important component of many neurodegenerative disorders, including acute ischemic stroke and chronic Parkinson’s disease. At the cellular level, microglia, the primary defender cells of the central nervous system, play an indispensable role in attenuating inflammation-initiated injury signals under physiological and pathological conditions [35]. Mechanistically, microglia release cytokines to reduce inflammation response and migrate to damaged tissue to participate in regenerative processes. However, chronic neuroinflammation reduces the viability of and promotes apoptosis in microglia, which in turn promotes the progression of neuroinflammation. Thus, inhibition of microglia death might be an effective treatment for neuroinflammation. In this study, we identified Sirt3 as a key upstream promoter of cell survival in microglia exposed to inflammation. Sirt3 expression was downregulated under inflammatory conditions, and restoration of Sirt3 levels significantly reduced the apoptotic rate in microglia by promoting mitochondrial functions and repressing mitochondrial apoptosis. Mechanistically, LPS triggered mitochondrial fission by increasing SRV2 expression; excessive fission then triggered caspase-9-assocaited mitochondrial apoptosis. In contrast, Sirt3 overexpression repressed SRV2-related mitochondrial fission and promoted survival in microglia under inflammation conditions by blocking the Mst1-JNK pathway. To our knowledge, this is the first study to describe the protective role of Sirt3 in neuroinflammation and the molecular mechanism responsible for its effects. Treatments that increase Sirt3 expression to inhibit the Mst1-JNK-SRV2-mitochondrial fission cascade might be highly effective in preventing neuroinflammation-induced microglia cell death.

Mitochondrial dysfunction has been associated with the progression of neuroinflammation [36]. For example, Aβ-mediated mitochondrial dysfunction contributes to the pathology of Alzheimer’s disease [37, 38]. Damaged mitochondria fail to produce sufficient ATP to maintain brain function and metabolism [39]. Additionally, mitochondrial dysfunction is always accompanied by overproduction of ROS, an early feature of oxidative stress. Uncontrolled oxidative stress induces cell senescence and promotes inflammation response [40]. Preservation of mitochondrial function via mitophagy [41] or pro-mitochondria drugs can be an effective treatment for neuroinflammation [42]. In this study, we also observed a reduction in mitochondrial function as a result of LPS stress. As was the case in previous studies, mitochondrial bioenergetics, redox balance, and survival were negatively affected by inflammation injury. Furthermore, our data illustrated that inflammation-related mitochondrial damage and mitochondrial apoptosis were largely the result of mitochondrial fission, which normally occurs in response to increases in metabolic rate. Abnormal mitochondrial fission, which results in the formation of non-functional mitochondrial debris, has been observed in response to stress-induced injury. This non-functional mitochondrial debris also acts as a source of pro-apoptotic proteins which eventually cause cell death. Our present results therefore suggest that inhibiting neuroinflammation-induced mitochondrial fission may help sustain mitochondrial homeostasis under LPS.

We also found that mitochondrial fission was inhibited by Sirt3, which agrees with previous studies. At the molecular level, Sirt3 inactivated the Mst1-JNK pathway and thus suppressed the expression of SRV2, a novel mediator of mitochondrial fission. The Mst1-JNK axis is known to control mitochondrial fission in liver cancer [43], hyperglycemia-induced vascular dysfunction [44], thyroid carcinoma [45], breast cancer [46], acute cardiac stress [47], and colorectal cancer [48]. In addition, SRV2 induces mitochondrial fission by promoting F-actin polymerization [49, 50]. Our data demonstrate that SRV2 is regulated by the Mst1-JNK pathway in LPS-treated BV-2 cells. We have thus identified a novel downstream effector of the Mst1-JNK pathway as well as a new molecular mechanism underlying neuroinflammation-induced mitochondrial fission.

In order to confirm the clinical relevance of these cell line experiment results, they should be replicated in studies using animal models and human samples [51]. Additional studies are also necessary to determine whether Sirt3 regulates other mitochondrial fission factors such as Drp1 and Mff in the context of neuroinflammation [52, 53]. Taken together, our results indicate that neuroinflammation-associated pathology is due at least in part to Sirt3 downregulation, which in turn activates the Mst1-JNK pathway and subsequent SRV2-related mitochondrial fission [54]. Moreover, overexpression of Sirt3 blocked the Mst1-JNK pathway and thus suppressed SRV2-induced mitochondrial fission, reducing inflammation-mediated mitochondrial dysfunction and microglia cell apoptosis. This suggests that Sirt3 might be an effective treatment for neuroinflammation.

## MATERIALS AND METHODS

### Cell culture

BV-2 cells obtained from ATCC (Rockefeller, USA) were cultured in Dulbecco’s modified Eagle’s medium (DMEM) (Gibco, C11995500CP) supplemented with 10% (v/v) fetal bovine serum (FBS) (BI, 04-001-1ACS) and 1% penicillin/streptomycin (Genom, GNM15140). All cells were maintained in a humidified incubator with 95% air and 5% CO_2_ at 37°C. For the inflammation injury model, BV-2 cells were incubated with LPS as previously described [55]. Additionally, anisomycin (Ani, 10 μM, Selleck Chemicals, Houston, TX, USA), an Mst1-JNK pathway agonist, was added to the BV-2 cell medium for 2 hours in order to activate the JNK pathway.

### Measurement of cell survival

Cell viability was determined using TUNEL, LDH release, and MTT assays. For the TUNEL assay, sections were deparaffinized and then incubated with proteinase K for 15 min at room temperature. Sections were then covered with TUNEL test solution containing fluorescein-conjugated dUTP and TdT enzyme at a ratio of 9:1 (v/v) in a humidified box at 37°C for 60 min. After washing, 4,6-diamidino-2-phenylindole (DAPI) was added to stain the cell nuclei. Images were taken using a fluorescent microscope (Olympus Corporation, Tokyo, Japan) [56, 57]. For LDH measurement, cellular medium samples were combined with 25 μL matrix buffer and 5 μL coenzyme I. After incubating for 15 min at 37°C, 25 μL 2,4-dinitrophenylhydrazine was added and incubated for another 15 min at 37°C. Finally, 250 μL 0.4mol/L NaOH was added to the mixture and incubated for 5 min, and optical density (OD) was measured using a microplate reader at 450 nm. For the MTT assay, 20 μL of MTT (5 mg/mL) in PBS solution was added to the medium after treatment and incubated for 4 h. The medium was then carefully removed and 150 μL of DMSO was added to each well to solubilize the crystals. Finally, OD was measured using a microplate reader at 490 nm [58].

### Measurement of MDA, GSH, and SOD levels

GPx, GSH, and SOD were quantified using commercial kits according to the manufacturer's protocol [59]. For GPx and SOD measurement, samples were rinsed with PBS and then homogenized and sonicated in lysis buffer on ice. After sonication, the lysed tissues were centrifuged at 10,000 g for 10 min to remove debris. For GSH measurement, samples were homogenized through three freeze-thaw cycles, and the tissue suspension was then centrifuged at 12,000 rpm for 5 min at 4°C. Supernatant GPx, GSH, and SOD levels were then measured. The protein concentration of each sample was determined using a BCA protein assay kit. In addition, GPx, GSH, and SOD levels were normalized to total protein concentrations [60].

### Cell fractionation and mitochondria isolation

BV-2 cells were plated in 10 cm dishes. Cytosolic and mitochondrial proteins were separated using a Cell Mitochondria Isolation Kit (Beyotime) according to the manufacturer’s instructions [61]. Briefly, cells were washed with pre-cooled PBS and lysed with Cell Mitochondria Isolation buffer on ice. Mitochondria and cytoplasm were separated by grinding followed by centrifugation at 600 g for 10 minutes at 4 °C. The supernatant was then further centrifuged at 11,000 g for 10 minutes at 4°C. The pellet was collected as the mitochondria-enriched fraction and resuspended in mitochondrial lysis buffer. The remaining supernatant was centrifuged 12,000 g for 10 minutes at 4°C to collect cytosolic proteins. Protein concentrations were detected using a Multimode Plate Reader (PerkinElmer). Equal amounts of protein (20 μg) from each fraction were measured by western blotting [62].

### Immunofluorescence microscopy

Following treatment, BV-2 cells on coverslips were fixed in 4% paraformaldehyde. The cells were then permeabilized and blocked with 2% goat serum containing 0.5% Triton X-100 and 3% BSA for 1 h at room temperature. The cells were probed with the following primary antibodies: Smac (1:1000, Cell Signaling Technology, #15108), Mst1 (1:1000, Cell Signaling Technology, #3682), and p-JNK (1:1,000; Cell Signaling Technology, #9251). After three PBS washes, cells were stained with secondary antibody. All fluorescent images were acquired on a confocal microscope (Olympus).

### Mitochondrial morphology assessment

Mitochondrial morphology was evaluated using MitoTracker Red (Invitrogen, USA) according to the manufacturer's instructions [63]. Briefly, the cells were incubated with 100 nM MitoTracker Red in RPMI 1640 medium for 30 min. Fluorescence was detected (490 nm excitation/525 nm emission) at 1000× magnification under a confocal laser scanning microscope (Olympus FV1200, Tokyo, Japan), and the images were analyzed using ImageJ (Bethesda, MD, USA). Mitochondrial fission evaluation was evaluated as described in previous studies [64, 65].

### Quantitative real-time PCR

mRNA was purified from cell pellets using the RNeasy Mini Kit (Qiagen #74104). mRNA was purified from EVs using the ExoRNeasy kit (Qiagen #77023). Up to 5 μg of total RNA were reverse-transcribed to obtain cDNA using SuperScript III (Invitrogen #18080-051). Quantitative PCR was performed using SYBR Green supermix (Bio-Rad #1725120). The manufacturers’ protocols were followed for all of these procedures [66]. GAPDH was used as the housekeeping gene. The following primers were used in the present study: Sirt3 (Forward: 5′-GGTGCCTAGTGAGAGTGAGTCCCC-3′ and Reverse: 5′-TCGGGGCTGAAGAGGGAGAA GTC-3′); GAPDH (Forward: 5′-ACGGCAAATTCAA CGGCACAGTCA-3′ and Reverse: 5′-TGGGGGCATC GGCAGAAGG-3′); Mff (Forward: 5′-AAGTGGCTCT CACCCTAGCA-3′ and Reverse: 5′-TGCCCCACTCA CCAAATGT-3′); Fis1 (Forward: 5′-CAAGGAACTGG AGCGGCTCATTA-3′ and Reverse: 5′- GGACACAG CAAGTCCGATGAGT-3′); Mfn1 (Forward: 5′-TGTG GTGGACTTCCTCTTGGC-3′ and Reverse: 5′-GAGA ATGAATGGGCGTGGG-3′); Mfn2, (Forward: 5′-AGG ATGACAATGGCATTGGC-3′ and Reverse: 5′-CCGATCGTACATCCGCTTAAC-3′).

### Measurement of mitochondrial ROS, mitochondrial membrane potential, and ATP production

Mitochondrial superoxide generation and membrane potential were measured as described previously [67]. ATP measurement kit (Beyotime, China) was used to measure ATP concentration as described previously [68]. Mitochondrial membrane potential was determined using the JC-1 probe (Beyotime, China). Red fluorescence from the JC-1 probe indicates normal mitochondrial membrane potential, whereas green fluorescence indicates abnormal mitochondrial membrane potential.

### Adenovirus transfection

BV-2 cells were transfected with Sirt3 and SRV2 adenovirus (Shanghai Gene-Pharma Co., Shanghai, China) according to the HiPerFect Transfection Reagent Handbook (QIAGEN). Briefly, BV-2 cells were washed with PBS after treatment and then infected with Sirt3 and/or SRV2 adenovirus for 48h using Lipofectamine 2000 (Invitrogen, 11668027) according to the manufacturer's specifications [69]. Subsequently, cells were isolated and overexpression efficiency was confirmed via qPCR.

### Protein extraction and western blot analysis

RIPA buffer was used to lyse the cells. Lysates were then centrifuged for 10 min at 12,000 rpm at 4°C. After quantification of protein concentration using BCA assay, equal amounts of protein were loaded on 10-12% gels for SDS-PAGE separation and then transferred onto PVDF membranes. After a 5% dry milk/TBST solution was used to block the membranes, primary antibodies, including rabbit IgG anti-LC3 (1:1000 dilution), and anti-GAPDH (1:5000 dilution) were added. After washing extensively with TBST, the goat anti-rabbit secondary antibody labeled with Alexa Fluor 680 (1:2000 dilution) was added. Membranes were scanned for far red signals using an Odyssey Imaging System (LI-COR, NE, USA). Quantity One Software (Bio-Rad, CA, USA) was used to quantify protein and expression levels by relative densitometry using beta-actin as the loading control.

### Statistical analysis

Student’s unpaired t-tests (two-group comparisons) and one-way ANOVAs (multigroup comparisons) were completed using GraphPad Prism. All data were analyzed to identify statistical significance between groups. P-values less than 0.05 were considered statistically significant, and the values are expressed as means ± SD.

## References

[1] Chen H, Kankel MW, Su SC, Han SW, Ofengeim D. Exploring the genetics and non-cell autonomous mechanisms underlying ALS/FTLD. Cell Death Differ. 2018; 25:648–62. 10.1038/s41418-018-0060-4 29459769 PMC5864209

[2] Lee K, Hwang OJ, Reiter RJ, Back K. Flavonoids inhibit both rice and sheep serotonin N-acetyltransferases and reduce melatonin levels in plants. J Pineal Res. 2018; 65:e12512. 10.1111/jpi.12512 29851162

[3] Burvenich IJ, Parakh S, Lee FT, Guo N, Liu Z, Gan HK, Rigopoulos A, O’Keefe GJ, Gong SJ, Goh YW, Tochon-Danguy H, Scott FE, Kotsuma M, et al. Molecular imaging of T cell co-regulator factor B7-H3 with ^89^Zr-DS-5573a. Theranostics. 2018; 8:4199–209. 10.7150/thno.25575 30128047 PMC6096400

[4] Wu Y, Yao J, Xie J, Liu Z, Zhou Y, Pan H, Han W. The role of autophagy in colitis-associated colorectal cancer. Signal Transduct Target Ther. 2018; 3:31. 10.1038/s41392-018-0031-8 30510778 PMC6265276

[5] Zhou H, Wang J, Hu S, Zhu H, Toanc S, Ren J. BI1 alleviates cardiac microvascular ischemia-reperfusion injury via modifying mitochondrial fission and inhibiting XO/ROS/F-actin pathways. J Cell Physiol. 2019; 234:5056–69. 10.1002/jcp.27308 30256421

[6] Darido C, Georgy SR, Cullinane C, Partridge DD, Walker R, Srivastava S, Roslan S, Carpinelli MR, Dworkin S, Pearson RB, Jane SM. Stage-dependent therapeutic efficacy in PI3K/mTOR-driven squamous cell carcinoma of the skin. Cell Death Differ. 2018; 25:1146–59. 10.1038/s41418-017-0032-0 29238073 PMC5988694

[7] Denton D, Kumar S. Autophagy-dependent cell death. Cell Death Differ. 2019; 26:605–16. 10.1038/s41418-018-0252-y 30568239 PMC6460387

[8] Zhou H, Li D, Zhu P, Hu S, Hu N, Ma S, Zhang Y, Han T, Ren J, Cao F, Chen Y. Melatonin suppresses platelet activation and function against cardiac ischemia/reperfusion injury via PPARγ/FUNDC1/mitophagy pathways. J Pineal Res. 2017; 63:63. 10.1111/jpi.12438 28749565

[9] Krause J, Löser A, Lemoine MD, Christ T, Scherschel K, Meyer C, Blankenberg S, Zeller T, Eschenhagen T, Stenzig J. Rat atrial engineered heart tissue: a new in vitro model to study atrial biology. Basic Res Cardiol. 2018; 113:41. 10.1007/s00395-018-0701-2 30178427

[10] Zhou H, Li D, Zhu P, Ma Q, Toan S, Wang J, Hu S, Chen Y, Zhang Y. Inhibitory effect of melatonin on necroptosis via repressing the Ripk3-PGAM5-CypD-mPTP pathway attenuates cardiac microvascular ischemia-reperfusion injury. J Pineal Res. 2018; 65:e12503. 10.1111/jpi.12503 29770487

[11] Yachi K, Tsuda M, Kohsaka S, Wang L, Oda Y, Tanikawa S, Ohba Y, Tanaka S. miR-23a promotes invasion of glioblastoma *via* HOXD10-regulated glial-mesenchymal transition. Signal Transduct Target Ther. 2018; 3:33. 10.1038/s41392-018-0033-6 30603114 PMC6308238

[12] Mehra P, Guo Y, Nong Y, Lorkiewicz P, Nasr M, Li Q, Muthusamy S, Bradley JA, Bhatnagar A, Wysoczynski M, Bolli R, Hill BG. Cardiac mesenchymal cells from diabetic mice are ineffective for cell therapy-mediated myocardial repair. Basic Res Cardiol. 2018; 113:46. 10.1007/s00395-018-0703-0 30353243 PMC6314032

[13] Zhou H, Zhang Y, Hu S, Shi C, Zhu P, Ma Q, Jin Q, Cao F, Tian F, Chen Y. Melatonin protects cardiac microvasculature against ischemia/reperfusion injury via suppression of mitochondrial fission-VDAC1-HK2-mPTP-mitophagy axis. J Pineal Res. 2017; 63:63. 10.1111/jpi.12413 28398674 PMC5518188

[14] Zhang M, Zhang Y, Xu E, Mohibi S, de Anda DM, Jiang Y, Zhang J, Chen X. Rbm24, a target of p53, is necessary for proper expression of p53 and heart development. Cell Death Differ. 2018; 25:1118–30. 10.1038/s41418-017-0029-8 29358667 PMC5988652

[15] Zhou H, Li N, Yuan Y, Jin YG, Guo H, Deng W, Tang QZ. Activating transcription factor 3 in cardiovascular diseases: a potential therapeutic target. Basic Res Cardiol. 2018; 113:37. 10.1007/s00395-018-0698-6 30094473

[16] He X, Zhang J, Li C, Zhang Y, Lu Y, Zhang Y, Liu L, Ruan C, Chen Q, Chen X, Guo Q, Sun T, Cheng J, Jiang C. Enhanced bioreduction-responsive diselenide-based dimeric prodrug nanoparticles for triple negative breast cancer therapy. Theranostics. 2018; 8:4884–97. 10.7150/thno.27581 30429875 PMC6217054

[17] Zhou H, Zhu P, Guo J, Hu N, Wang S, Li D, Hu S, Ren J, Cao F, Chen Y. Ripk3 induces mitochondrial apoptosis via inhibition of FUNDC1 mitophagy in cardiac IR injury. Redox Biol. 2017; 13:498–507. 10.1016/j.redox.2017.07.007 28732308 PMC5828768

[18] Fisher AB, Vasquez-Medina JP, Dodia C, Sorokina EM, Tao JQ, Feinstein SI. Peroxiredoxin 6 phospholipid hydroperoxidase activity in the repair of peroxidized cell membranes. Redox Biol. 2018; 14:41–46. 10.1016/j.redox.2017.08.008 28865296 PMC5581854

[19] Pérez-González A, Castañeda-Arriaga R, Álvarez-Idaboy JR, Reiter RJ, Galano A. Melatonin and its metabolites as chemical agents capable of directly repairing oxidized DNA. J Pineal Res. 2019; 66:e12539. 10.1111/jpi.12539 30417425

[20] Jin Q, Li R, Hu N, Xin T, Zhu P, Hu S, Ma S, Zhu H, Ren J, Zhou H. DUSP1 alleviates cardiac ischemia/reperfusion injury by suppressing the Mff-required mitochondrial fission and Bnip3-related mitophagy via the JNK pathways. Redox Biol. 2018; 14:576–87. 10.1016/j.redox.2017.11.004 29149759 PMC5691221

[21] Zhou H, Wang S, Zhu P, Hu S, Chen Y, Ren J. Empagliflozin rescues diabetic myocardial microvascular injury via AMPK-mediated inhibition of mitochondrial fission. Redox Biol. 2018; 15:335–46. 10.1016/j.redox.2017.12.019 29306791 PMC5756062

[22] Chen YC, Cheng TH, Lin WL, Chen CL, Yang WY, Blackstone C, Chang CR. Srv2 Is a Pro-fission Factor that Modulates Yeast Mitochondrial Morphology and Respiration by Regulating Actin Assembly. iScience. 2019; 11:305–317. 10.1016/j.isci.2018.12.021 30639852 PMC6327880

[23] Lee HJ, Kang MG, Cha HY, Kim YM, Lim Y, Yang SJ. Effects of Piceatannol and Resveratrol on Sirtuins and Hepatic Inflammation in High-Fat Diet-Fed Mice. J Med Food. 2019; 22:833–40. 10.1089/jmf.2018.4261 31268397

[24] Boniakowski AM, denDekker AD, Davis FM, Joshi A, Kimball AS, Schaller M, Allen R, Bermick J, Nycz D, Skinner ME, Robinson S, Obi AT, Moore BB, et al. SIRT3 Regulates Macrophage-Mediated Inflammation in Diabetic Wound Repair. J Invest Dermatol. 2019. [Epub ahead of print]. 10.1016/j.jid.2019.05.017 31207226 PMC7185380

[25] Pu J, Zhu S, Zhou D, Zhao L, Yin M, Wang Z, Hong J. Propofol Alleviates Apoptosis Induced by Chronic High Glucose Exposure via Regulation of HIF-1*α* in H9c2 Cells. Oxid Med Cell Longev. 2019; 2019:4824035. 10.1155/2019/4824035 31093315 PMC6481038

[26] Wang Y, Chen Y, Guan L, Zhang H, Huang Y, Johnson CH, Wu Z, Gonzalez FJ, Yu A, Huang P, Wang Y, Yang S, Chen P, et al. Carnitine palmitoyltransferase 1C regulates cancer cell senescence through mitochondria-associated metabolic reprograming. Cell Death Differ. 2018; 25:735–48. 10.1038/s41418-017-0013-3 29317762 PMC5864250

[27] Huang D, Liu M, Jiang Y. Mitochonic acid-5 attenuates TNF-α-mediated neuronal inflammation via activating Parkin-related mitophagy and augmenting the AMPK-Sirt3 pathways. J Cell Physiol. 2019; 234:22172–82. 10.1002/jcp.28783 31062359

[28] Lan S, Liu J, Luo X, Bi C. Effects of melatonin on acute brain reperfusion stress: role of Hippo signaling pathway and MFN2-related mitochondrial protection. Cell Stress Chaperones. 2019; 24:235–45. 10.1007/s12192-018-00960-2 30632064 PMC6363627

[29] Chen Y, Liu K, Shi Y, Shao C. The tango of ROS and p53 in tissue stem cells. Cell Death Differ. 2018; 25:639–41. 10.1038/s41418-018-0062-2 29487352 PMC5864234

[30] Zhao Z, Lu C, Li T, Wang W, Ye W, Zeng R, Ni L, Lai Z, Wang X, Liu C. The protective effect of melatonin on brain ischemia and reperfusion in rats and humans: in vivo assessment and a randomized controlled trial. J Pineal Res. 2018; 65:e12521. 10.1111/jpi.12521 30098076

[31] Zhou YQ, Liu DQ, Chen SP, Sun J, Zhou XR, Rittner H, Mei W, Tian YK, Zhang HX, Chen F, Ye DW. Reactive oxygen species scavengers ameliorate mechanical allodynia in a rat model of cancer-induced bone pain. Redox Biol. 2018; 14:391–97. 10.1016/j.redox.2017.10.011 29055283 PMC5650652

[32] Hu S, Zhu P, Zhou H, Zhang Y, Chen Y. Melatonin-Induced Protective Effects on Cardiomyocytes Against Reperfusion Injury Partly Through Modulation of IP3R and SERCA2a Via Activation of ERK1. Arq Bras Cardiol. 2018; 110:44–51. 10.5935/abc.20180008 29538523 PMC5831301

[33] Olson KR, Gao Y, Arif F, Arora K, Patel S, DeLeon ER, Sutton TR, Feelisch M, Cortese-Krott MM, Straub KD. Metabolism of hydrogen sulfide (H_2_S) and Production of Reactive Sulfur Species (RSS) by superoxide dismutase. Redox Biol. 2018; 15:74–85. 10.1016/j.redox.2017.11.009 29220697 PMC5725220

[34] Zhou H, Wang J, Zhu P, Zhu H, Toan S, Hu S, Ren J, Chen Y. NR4A1 aggravates the cardiac microvascular ischemia reperfusion injury through suppressing FUNDC1-mediated mitophagy and promoting Mff-required mitochondrial fission by CK2α. Basic Res Cardiol. 2018; 113:23. 10.1007/s00395-018-0682-1 29744594

[35] Zhou H, Zhu P, Wang J, Zhu H, Ren J, Chen Y. Pathogenesis of cardiac ischemia reperfusion injury is associated with CK2α-disturbed mitochondrial homeostasis via suppression of FUNDC1-related mitophagy. Cell Death Differ. 2018; 25:1080–93. 10.1038/s41418-018-0086-7 29540794 PMC5988750

[36] Hao L, Sun Q, Zhong W, Zhang W, Sun X, Zhou Z. Mitochondria-targeted ubiquinone (MitoQ) enhances acetaldehyde clearance by reversing alcohol-induced posttranslational modification of aldehyde dehydrogenase 2: A molecular mechanism of protection against alcoholic liver disease. Redox Biol. 2018; 14:626–36. 10.1016/j.redox.2017.11.005 29156373 PMC5700831

[37] Shin SJ, Jeon SG, Kim JI, Jeong YO, Kim S, Park YH, Lee SK, Park HH, Hong SB, Oh S, Hwang JY, Kim HS, Park H, et al. Red Ginseng Attenuates Aβ-Induced Mitochondrial Dysfunction and Aβ-mediated Pathology in an Animal Model of Alzheimer’s Disease. Int J Mol Sci. 2019; 20:20. 10.3390/ijms20123030 31234321 PMC6627470

[38] Zhou H, Yue Y, Wang J, Ma Q, Chen Y. Melatonin therapy for diabetic cardiomyopathy: A mechanism involving Syk-mitochondrial complex I-SERCA pathway. Cell Signal. 2018; 47:88–100. 10.1016/j.cellsig.2018.03.012 29601906

[39] Bader V, Winklhofer KF. Mitochondria at the interface between neurodegeneration and neuroinflammation. Semin Cell Dev Biol. 2019. [Epub ahead of print]. 10.1016/j.semcdb.2019.05.028 31154011

[40] Bajwa E, Pointer CB, Klegeris A. The Role of Mitochondrial Damage-Associated Molecular Patterns in Chronic Neuroinflammation. Mediators Inflamm. 2019; 2019:4050796. 10.1155/2019/4050796 31065234 PMC6466851

[41] Lautrup S, Lou G, Aman Y, Nilsen H, Tao J, Fang EF. Microglial mitophagy mitigates neuroinflammation in Alzheimer’s disease. Neurochem Int. 2019; 129:104469. 10.1016/j.neuint.2019.104469 31100304

[42] Zhou H, Wang J, Zhu P, Hu S, Ren J. Ripk3 regulates cardiac microvascular reperfusion injury: the role of IP3R-dependent calcium overload, XO-mediated oxidative stress and F-action/filopodia-based cellular migration. Cell Signal. 2018; 45:12–22. 10.1016/j.cellsig.2018.01.020 29413844

[43] Wang A, Wang J, Wu J, Deng X, Zou Y. Suramin protects hepatocytes from LPS-induced apoptosis by regulating mitochondrial stress and inactivating the JNK-Mst1 signaling pathway. J Physiol Sci. 2019; 69:489–502. 10.1007/s12576-019-00666-9 30771091 PMC10717776

[44] Tian H, Wang K, Jin M, Li J, Yu Y. Proinflammation effect of Mst1 promotes BV-2 cell death via augmenting Drp1-mediated mitochondrial fragmentation and activating the JNK pathway. J Cell Physiol. 2019. [Epub ahead of print]. 10.1002/jcp.29070 31283035

[45] Zhang X, Li F, Cui Y, Liu S, Sun H. Mst1 overexpression combined with Yap knockdown augments thyroid carcinoma apoptosis via promoting MIEF1-related mitochondrial fission and activating the JNK pathway. Cancer Cell Int. 2019; 19:143. 10.1186/s12935-019-0860-8 31139020 PMC6530088

[46] Ouyang H, Zhou E, Wang H. Mst1-Hippo pathway triggers breast cancer apoptosis via inducing mitochondrial fragmentation in a manner dependent on JNK-Drp1 axis. Onco Targets Ther. 2019; 12:1147–59. 10.2147/OTT.S193787 30809096 PMC6376886

[47] Cheng Z, Zhang M, Hu J, Lin J, Feng X, Wang S, Wang T, Gao E, Wang H, Sun D. Cardiac-specific Mst1 deficiency inhibits ROS-mediated JNK signalling to alleviate Ang II-induced cardiomyocyte apoptosis. J Cell Mol Med. 2019; 23:543–55. 10.1111/jcmm.13958 30338935 PMC6307828

[48] Li Q, Qi F, Meng X, Zhu C, Gao Y. Mst1 regulates colorectal cancer stress response via inhibiting Bnip3-related mitophagy by activation of JNK/p53 pathway. Cell Biol Toxicol. 2018; 34:263–77. 10.1007/s10565-017-9417-6 29063978

[49] Zhu H, Jin Q, Li Y, Ma Q, Wang J, Li D, Zhou H, Chen Y. Melatonin protected cardiac microvascular endothelial cells against oxidative stress injury via suppression of IP3R-[Ca^2+^]c/VDAC-[Ca^2+^]m axis by activation of MAPK/ERK signaling pathway. Cell Stress Chaperones. 2018; 23:101–13. 10.1007/s12192-017-0827-4 28669047 PMC5741585

[50] Zhou H, Wang S, Hu S, Chen Y, Ren J. ER-Mitochondria Microdomains in Cardiac Ischemia-Reperfusion Injury: A Fresh Perspective. Front Physiol. 2018; 9:755. 10.3389/fphys.2018.00755 29962971 PMC6013587

[51] Álvarez-Fernández M, Sanz-Flores M, Sanz-Castillo B, Salazar-Roa M, Partida D, Zapatero-Solana E, Ali HR, Manchado E, Lowe S, VanArsdale T, Shields D, Caldas C, Quintela-Fandino M, Malumbres M. Therapeutic relevance of the PP2A-B55 inhibitory kinase MASTL/Greatwall in breast cancer. Cell Death Differ. 2018; 25:828–40. 10.1038/s41418-017-0024-0 29229993 PMC5943447

[52] Anderton H, Bandala-Sanchez E, Simpson DS, Rickard JA, Ng AP, Di Rago L, Hall C, Vince JE, Silke J, Liccardi G, Feltham R. RIPK1 prevents TRADD-driven, but TNFR1 independent, apoptosis during development. Cell Death Differ. 2019; 26:877–89. 10.1038/s41418-018-0166-8 30185824 PMC6461919

[53] Zhou H, Hu S, Jin Q, Shi C, Zhang Y, Zhu P, Ma Q, Tian F, Chen Y. Mff-Dependent Mitochondrial Fission Contributes to the Pathogenesis of Cardiac Microvasculature Ischemia/Reperfusion Injury via Induction of mROS-Mediated Cardiolipin Oxidation and HK2/VDAC1 Disassociation-Involved mPTP Opening. J Am Heart Assoc. 2017; 6:6. 10.1161/JAHA.116.005328 28288978 PMC5524036

[54] Avalle L, Camporeale A, Morciano G, Caroccia N, Ghetti E, Orecchia V, Viavattene D, Giorgi C, Pinton P, Poli V. STAT3 localizes to the ER, acting as a gatekeeper for ER-mitochondrion Ca^2+^ fluxes and apoptotic responses. Cell Death Differ. 2019; 26:932–42. 10.1038/s41418-018-0171-y 30042492 PMC6214529

[55] Armartmuntree N, Murata M, Techasen A, Yongvanit P, Loilome W, Namwat N, Pairojkul C, Sakonsinsiri C, Pinlaor S, Thanan R. Prolonged oxidative stress down-regulates Early B cell factor 1 with inhibition of its tumor suppressive function against cholangiocarcinoma genesis. Redox Biol. 2018; 14:637–44. 10.1016/j.redox.2017.11.011 29169115 PMC5701798

[56] Coverstone ED, Bach RG, Chen L, Bierut LJ, Li AY, Lenzini PA, O’Neill HC, Spertus JA, Sucharov CC, Stitzel JA, Schilling JD, Cresci S. A novel genetic marker of decreased inflammation and improved survival after acute myocardial infarction. Basic Res Cardiol. 2018; 113:38. 10.1007/s00395-018-0697-7 30097758 PMC6292447

[57] Zhou H, Shi C, Hu S, Zhu H, Ren J, Chen Y. BI1 is associated with microvascular protection in cardiac ischemia reperfusion injury via repressing Syk-Nox2-Drp1-mitochondrial fission pathways. Angiogenesis. 2018; 21:599–615. 10.1007/s10456-018-9611-z 29623489

[58] Zhu P, Hu S, Jin Q, Li D, Tian F, Toan S, Li Y, Zhou H, Chen Y. Ripk3 promotes ER stress-induced necroptosis in cardiac IR injury: A mechanism involving calcium overload/XO/ROS/mPTP pathway. Redox Biol. 2018; 16:157–68. 10.1016/j.redox.2018.02.019 29502045 PMC5952878

[59] Shi C, Cai Y, Li Y, Li Y, Hu N, Ma S, Hu S, Zhu P, Wang W, Zhou H. Yap promotes hepatocellular carcinoma metastasis and mobilization via governing cofilin/F-actin/lamellipodium axis by regulation of JNK/Bnip3/SERCA/CaMKII pathways. Redox Biol. 2018; 14:59–71. 10.1016/j.redox.2017.08.013 28869833 PMC5582718

[60] Batista IA, Helguero LA. Biological processes and signal transduction pathways regulated by the protein methyltransferase SETD7 and their significance in cancer. Signal Transduct Target Ther. 2018; 3:19. 10.1038/s41392-018-0017-6 30013796 PMC6043541

[61] Aanhane E, Schulkens IA, Heusschen R, Castricum K, Leffler H, Griffioen AW, Thijssen VL. Different angioregulatory activity of monovalent galectin-9 isoforms. Angiogenesis. 2018; 21:545–55. 10.1007/s10456-018-9607-8 29500586

[62] Li R, Xin T, Li D, Wang C, Zhu H, Zhou H. Therapeutic effect of Sirtuin 3 on ameliorating nonalcoholic fatty liver disease: the role of the ERK-CREB pathway and Bnip3-mediated mitophagy. Redox Biol. 2018; 18:229–43. 10.1016/j.redox.2018.07.011 30056271 PMC6079484

[63] Zou Y, Wei J, Xia Y, Meng F, Yuan J, Zhong Z. Targeted chemotherapy for subcutaneous and orthotopic non-small cell lung tumors with cyclic RGD-functionalized and disulfide-crosslinked polymersomal doxorubicin. Signal Transduct Target Ther. 2018; 3:32. 10.1038/s41392-018-0032-7 30564464 PMC6292884

[64] Deussen A. Mechanisms underlying coronary autoregulation continue to await clarification. Basic Res Cardiol. 2018; 113:34. 10.1007/s00395-018-0693-y 30074094

[65] Capece D, D’Andrea D, Verzella D, Tornatore L, Begalli F, Bennett J, Zazzeroni F, Franzoso G. Turning an old GADDget into a troublemaker. Cell Death Differ. 2018; 25:642–44. 10.1038/s41418-018-0087-6 29511335 PMC5864189

[66] Chandra M, Escalante-Alcalde D, Bhuiyan MS, Orr AW, Kevil C, Morris AJ, Nam H, Dominic P, McCarthy KJ, Miriyala S, Panchatcharam M. Cardiac-specific inactivation of LPP3 in mice leads to myocardial dysfunction and heart failure. Redox Biol. 2018; 14:261–71. 10.1016/j.redox.2017.09.015 28982073 PMC5635346

[67] Crist AM, Lee AR, Patel NR, Westhoff DE, Meadows SM. Vascular deficiency of Smad4 causes arteriovenous malformations: a mouse model of Hereditary Hemorrhagic Telangiectasia. Angiogenesis. 2018; 21:363–80. 10.1007/s10456-018-9602-0 29460088 PMC5878194

[68] Zhou X, Li X, Wu M. miRNAs reshape immunity and inflammatory responses in bacterial infection. Signal Transduct Target Ther. 2018; 3:14. 10.1038/s41392-018-0006-9 29844933 PMC5968033

[69] Fan T, Pi H, Li M, Ren Z, He Z, Zhu F, Tian L, Tu M, Xie J, Liu M, Li Y, Tan M, Li G, et al. Inhibiting MT2-TFE3-dependent autophagy enhances melatonin-induced apoptosis in tongue squamous cell carcinoma. J Pineal Res. 2018; 64:64. 10.1111/jpi.12457 29149494

